# Semi-Lethal Primary Ciliary Dyskinesia in Rats Lacking the *Nme7* Gene

**DOI:** 10.3390/ijms22083810

**Published:** 2021-04-07

**Authors:** Lucie Šedová, Ivana Buková, Pavla Bažantová, Silvia Petrezsélyová, Jan Prochazka, Elena Školníková, Dagmar Zudová, Josef Včelák, Pavol Makovický, Běla Bendlová, Ondřej Šeda, Radislav Sedlacek

**Affiliations:** 1 Laboratory of Transgenic Models of Diseases, Institute of Molecular Genetics of the Czech Academy of Sciences, v.v.i., 252 50 Vestec, Czech Republic; silvia.petrezselyova@img.cas.cz (S.P.); elena.skolnikova@gmail.com (E.Š.); radislav.sedlacek@img.cas.cz (R.S.); 2 Institute of Biology and Medical Genetics, The First Faculty of Medicine, Charles University and the General University Hospital, 128 00 Prague, Czech Republic; pavla.bazantova@lf1.cuni.cz (P.B.); oseda@lf1.cuni.cz (O.Š.); 3 Czech Centre for Phenogenomics, Institute of Molecular Genetics of the Czech Academy of Sciences, v.v.i., 252 50 Vestec, Czech Republic; ivana.bukova@img.cas.cz (I.B.); jan.prochazka@img.cas.cz (J.P.); dagmar.zudova@img.cas.cz (D.Z.); 4 Department of Molecular Endocrinology, Institute of Endocrinology, 116 94 Prague, Czech Republic; JVcelak@endo.cz (J.V.); bbendlova@endo.cz (B.B.); 5 Department of Biology, Faculty of Education, J. Selye University, 945 01 Komarno, Slovakia; makovickyp@ujs.sk

**Keywords:** cilia, hydrocephalus, Nme7, knock-out rat, infertility

## Abstract

*NME7* (non-metastatic cells 7, nucleoside diphosphate kinase 7) is a member of a gene family with a profound effect on health/disease status. NME7 is an established member of the ciliome and contributes to the regulation of the microtubule-organizing center. We aimed to create a rat model to further investigate the phenotypic consequences of *Nme7* gene deletion. The CRISPR/Cas9 nuclease system was used for the generation of Sprague Dawley *Nme7* knock-out rats targeting the exon 4 of the *Nme7* gene. We found the homozygous *Nme7* gene deletion to be semi-lethal, as the majority of SD*^Nme7^*^−/−^ pups died prior to weaning. The most prominent phenotypes in surviving SD*^Nme7^*^−/−^ animals were hydrocephalus, situs inversus totalis, postnatal growth retardation, and sterility of both sexes. Thinning of the neocortex was histologically evident at 13.5 day of gestation, dilation of all ventricles was detected at birth, and an external sign of hydrocephalus, i.e., doming of the skull, was usually apparent at 2 weeks of age. Heterozygous SD*^Nme7^*^+/−^ rats developed normally; we did not detect any symptoms of primary ciliary dyskinesia. The transcriptomic profile of liver and lungs corroborated the histological findings, revealing defects in cell function and viability. In summary, the knock-out of the rat *Nme7* gene resulted in a range of conditions consistent with the presentation of primary ciliary dyskinesia, supporting the previously implicated role of the centrosomally located *Nme7* gene in ciliogenesis and control of ciliary transport.

## 1. Introduction

Primary ciliary dyskinesia is a heterogeneous disorder affecting motile cilia and primarily manifests in humans as chronic sinusitis, bronchiectasis, and situs inversus (known as Kartagener syndrome) [1]. Additional clinical features may comprise male infertility, congenital heart defects, hydrocephalus, female subfertility, and others. Genes so far associated with primary ciliary dyskinesia mainly encode cilia structure components, but genes with regulatory and other functions have also been reported [2]. The NME/NM23 family of human nucleoside diphosphate kinase multifunctional proteins comprises 10 members in humans, with a distinct extent of enzymatic activities and tissue and cellular distribution patterns [3,4]. One of its members, NME7 (NME/NM23 family member 7), shows an almost ubiquitous expression and belongs phylogenetically to a group of NME proteins with low or no nucleoside-diphosphate kinase activity [3,5]. NME7 was implicated in the regulation of the microtubule-nucleating activity of the γ-tubulin ring complex in centrosomes connected with the biogenesis and function of motile cilia [5,6]. However, several lines of evidence point to a multitude of physiological processes in which NME7 is involved. It was reported as an essential factor for stem cell renewal [7] and maintenance of early stem cells in the naive status [8]. The potential broader functional outreach of NME7 is also supported by the results of several genome-wide association studies, connecting the variation in the human *Nme7* gene with venous thromboembolism [9,10,11], coronary artery disease [12], systolic blood pressure, pulse pressure [12,13], or QT interval [14,15]. The strains with the lowest hepatic *Nme7* expression in a genetically designed rat model panel showed perturbations of transcriptomic modules related to ciliogenesis, and carbohydrate and lipid metabolism [16]. In the *Nme7* knock-out mice model and the reported human *Nme7* homozygous mutation carriers, hydrocephalus and situs inversus were the most apparent features [17,18,19]. However, a detailed account of phenotypic characterization across other systems is missing. In the current study, we aimed to generate an *Nme7*-deficient rat model and characterize the effect of *Nme7* loss on the morphological and transcriptomic levels.

## 2. Results

### 2.1. Generation of an Nme7 Knock-Out Rat Model

**Figure 1:**
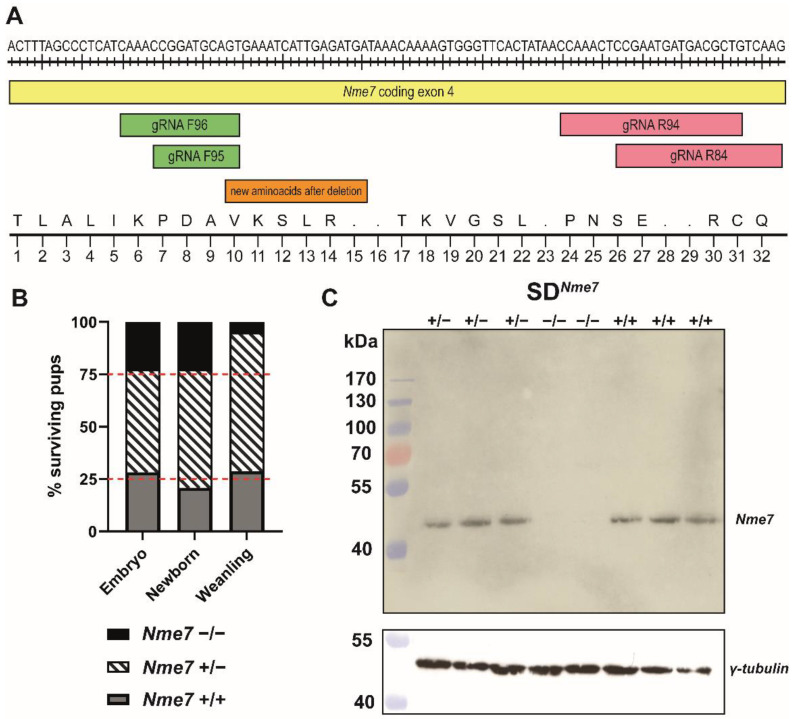
Generation of an *Nme7* knock-out Sprague Dawley rat model. (**A**) At the top, part of the sequence of *Nme7*, coding exon 4, is shown together with the locations of two sets of guide (g)RNAs. The bottom part of the figure shows the corresponding amino acid sequence (standard one-letter code), indicating its change after the induced deletion. (**B**) Ratio of the genotypes of surviving SD*^Nme7^* rats at three distinct time points (embryo: E13.5–E15.5, newborn: P1, and weanling: P28). The red dashed line corresponds to the expected Mendelian ratio of 25% (SD*^Nme7+/+^*—grey bars):50% (SD*^Nme7^*^+/−^—striped bars):25% (SD*^Nme7^*^−/−^—black bars). (**C**) Western blot showing the lack of Nme7 protein in SD*^Nme7^*^−/−^ rat testes. Stripped membrane was used for γ-tubulin protein detection.

The CRISPR/Cas9 nuclease system was used to generate Sprague Dawley (SD) rats with *Nme7* gene knock-out. Two sets of guide (g)RNA were designed within the exon 4 of the *Nme7* gene. Using this strategy, we were able to produce and verify a knock-out founder with a 5-nucleotide deletion (TCGAA within the exon 4 of the *Nme7* gene) responsible for creating a premature stop codon (Figure 1A).

The founder was bred with wild-type Sprague Dawley rats, and only offspring carrying the 5-nucleotide deletion were selected for further breeding with wild-type SD rats. After three consecutive generations, the heterozygotes carrying the 5-nucleotide deletion were sequenced and then intercrossed to establish the *Nme7* knock-out line on SD background (SD*^Nme7^*). During the breeding of the SD*^Nme7^* line, we had 36 litters with an average number of 9.7 newborns per litter. The distribution of SD*^Nme7−/−^* vs. SD*^Nme7+/−^* vs. SD genotypes at birth followed the Mendelian laws for mating of heterozygous animals, i.e., 1:2:1, but soon after birth, the SD*^Nme7−/−^* animals started to show external signs of hydrocephalus (doming of the skull) and began to prosper poorly, and less than one-quarter of them survived till weaning at the age of 4 weeks (Figure 1B).

### 2.2. Morphometric Assessment of SD*^Nme7−/−^* Rats

**Figure 2:**
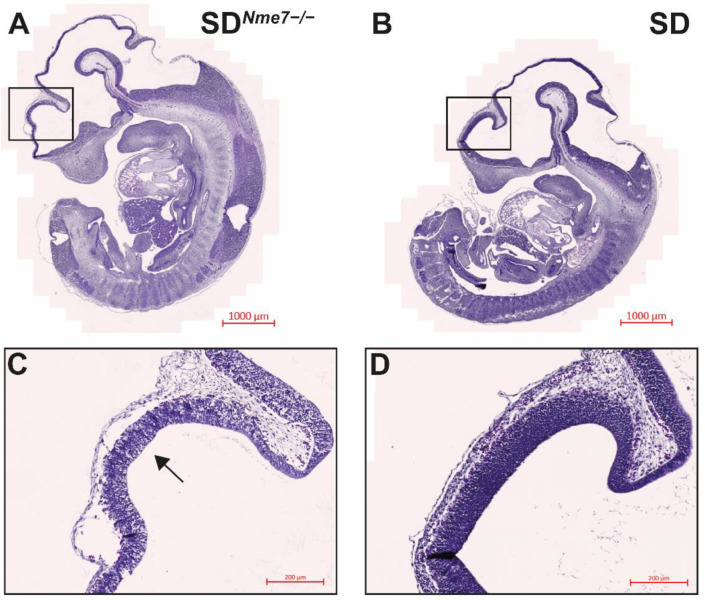
Hematoxylin–eosin-stained mid-sagittal sections of (**A**) SD*^Nme7^*^−/−^ and (**B**) SD embryos at E13.5. Blow-up images (**C**,**D**) show differences in the developing brain cortex. The SD*^Nme7^*^−/−^ rat embryo shows thinner ventricular zone (arrow) of neopallial cortex, with reduced cellularity in both cortical and thalamic regions. The bars indicate 1000 µm for (**A**,**B**) and 200 µm for (**C**,**D**).

**Figure 3:**
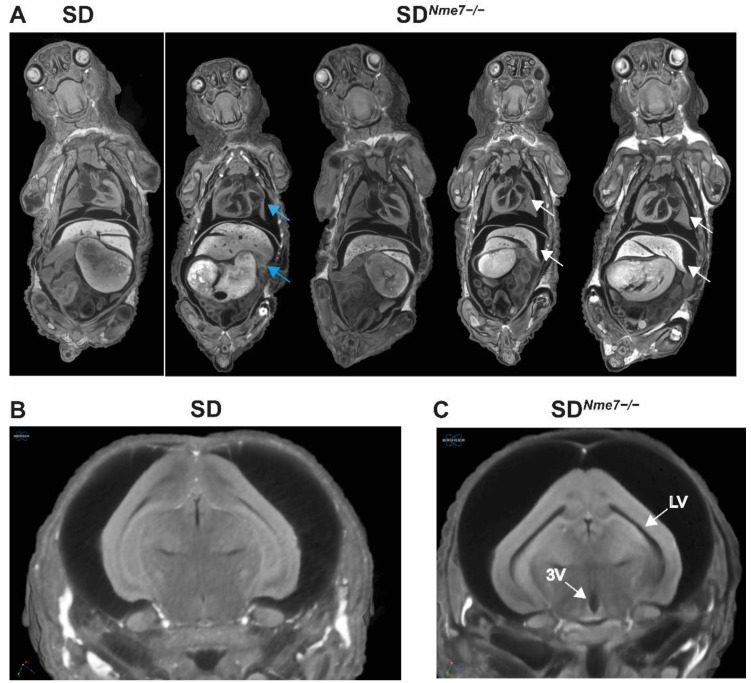
Micro-CT showing (**A**) situs inversus totalis with dextrocardia (white arrows) and 90° rotation of inner organs (blue arrows) in SD*^Nme7^*^−/−^ rats in comparison with SD control rat (all P1). Detailed view of enlarged lateral ventricles (LV) and 3rd ventricle (3V) in brains of SD*^Nme7^*^−/−^ rats (**C**) compared to SD rats (**B**).

To examine the effects of *Nme7* on rat embryonic development, we analyzed embryos at embryonic day (E) 13.5, 14.5, and 15.5. We did not observe any size differences between SD*^Nme7^*^−/−^ and SD or heterozygous SD*^Nme7^*^+/−^ embryos assessed by the crown-to-rump length measurement (Figure S1). However, histological analysis of mutant embryos revealed atrophy of neopallial cortex, with thinner ventricular zone and reduced cellularity in both cortex and thalamus already at E13.5 (Figure 2). At birth, SD*^Nme7^*^−/−^ rats did not show any distinct external features compared to their siblings. Micro-CT scan performed at postnatal day 1 (P1) revealed random left/right symmetry dysregulation with situs inversus totalis present in most SD*^Nme7^*^−/−^ rats, manifested as complete transposition of the abdominal organs and dextrocardia (Figure 3). Dilation of all brain ventricles, thinning of the cortex, and reduction of the cerebellum due to compression were major alterations of brain structure in SD*^Nme7^*^−/−^ rats (Figure 3). Two-week old SD*^Nme7^*^−/−^ rats already presented external manifestations of the *Nme7*^−/−^ phenotype, including domed skull, slower and less coordinated movements, and smaller body size (Supplementary Video S1). The postnatal growth restriction progressed, as at 6 weeks of age, the body weights were: SD*^Nme7^*^−/−^, 131 ± 37 g; SD*^Nme7^*^+/−^, 239 ± 61 g; SD, 236 ± 55 g, P_ANOVA_ = 0.0002, Tukey’s HSD post-hoc *p* = 0.0001 and *p* = 0.0005 for SD*^Nme7^*^−/−^ vs. SD*^Nme7^*^+/−^ and SD*^Nme7^*^−/−^ vs. SD, respectively. Heart, kidney, liver, spleen, and testes weights per 100 g of body weight were comparable between SD*^Nme7^*^−/−^ and SD rats.

### 2.3. Histological Assessment of SD*^Nme7−/−^* Rats

**Figure 4:**
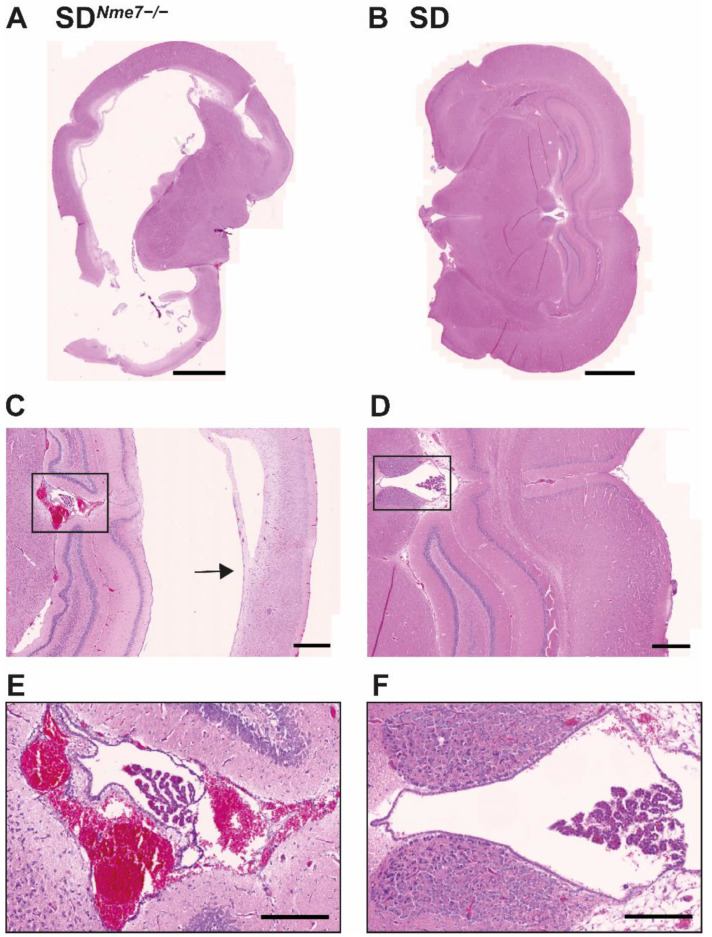
Coronal section of hematoxylin–eosin-stained brain of 6-week old SD*^Nme7−/−^* rat (**A**) showing massive hydrocephalus with greatly enlarged lateral ventricles compared to SD littermate (**B**). Detail of deformed hippocampal region with thinning of adjacent cortex (arrow) in the SD*^Nme7−/−^* rat (**C**) compared to the SD counterpart (**D**). Blow-up of the third ventricle with massive bleeding, damaged choroid plexus and ependymal lining in SD*^Nme7−/−^* rat (**E**) contrasting with the standard arrangement of the third ventricle in the SD rat (**F**). The bar indicates 2000 µm for (**A**,**B**), 500 µm for (**C**,**D**), 200 µm for (**E**,**F**).

Hydrocephalus was detected in all SD*^Nme7−/−^* rats. Transversally sectioned SD*^Nme7−/−^* rat brains showed massively dilated lateral ventricles (Figure 4A,B). We observed an overall disintegration of brain architecture, massive bleeding, loss of brain layering, and cortical atrophy (Figure 4C,D). The ependymal lining of the ventricles as well as the choroid plexi were damaged (Figure 4E,F). Subarachnoid spaces were also dilated with signs of old and new hemorrhages.

**Figure 5:**
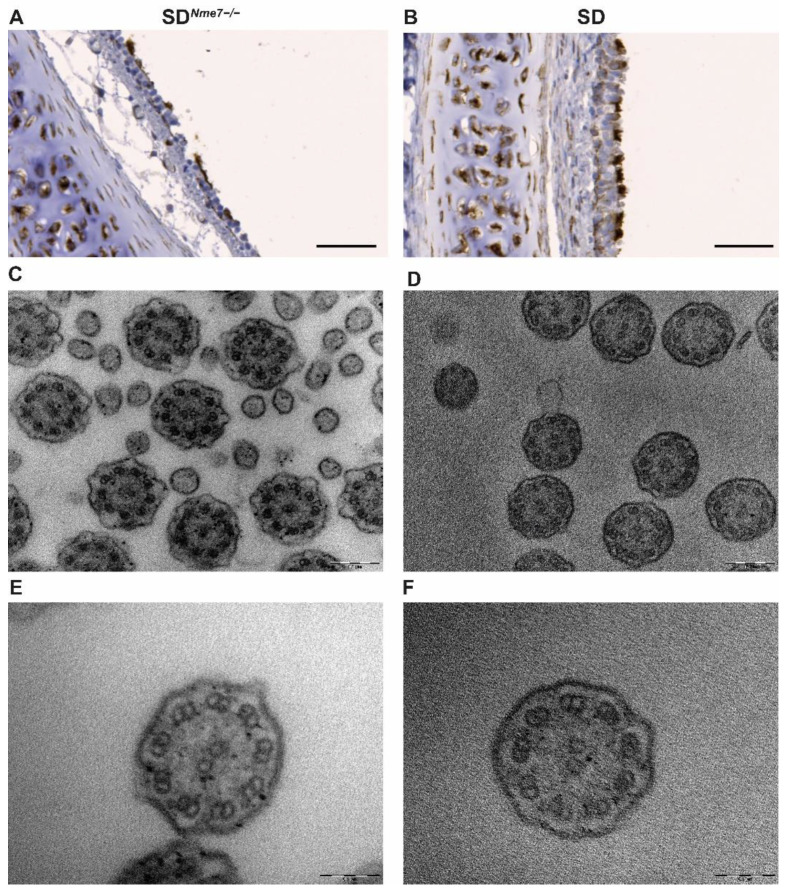
Acetylated α-tubulin staining in transversally sectioned trachea ((**A**,**B**), the bar indicates 50 µm). Cilia staining is evident in both SD*^Nme7−/−^* (**A**) and SD control rats (**B**), even though the pseudostratified cilliated columnar epithelium is damaged and, in some parts, even missing in SD*^Nme7−/−^* rats. Transmission electron microscopy of cilia in the trachea (magnification 56,000× ((**C**,**D**), the bar indicates 0.2 µm) and 140,000× ((**E**,**F**), the bar indicates 0.1 µm)) shows standard arrangement of the cilia ultrastructure on the cross-sectional view—nine outer doublets and one central pair (9 × 2 + 2) both in SD*^Nme7−/−^* (**C**,**E**) and in SD control rats (**D**,**F**).

The trachea was lined by a ciliated pseudostratified epithelium that showed strong Nme7 positivity in SD rats compared to a disrupted lining of the trachea with scarce cilia in SD*^Nme7−/−^* rats (Figure S2). Cilia on the ciliated pseudostratified epithelium of the trachea were examined immunohistologically using acetylated α-tubulin staining (Figure 5A,B) and by transmission electron microscopy. A cross section of SD*^Nme7−/−^* cilia did not reveal any major structural changes compared to a cross-section of SD cilia (Figure 5C–F).

Rhinitis and sinusitis, evident as serous or mucinous deposits, were present in all examined SD*^Nme7−/−^* rats (Figure S3). The lungs were also affected in SD*^Nme7−/−^* rats; the alveolar lumen contained serous secretion and blood, the ciliated pseudostratified columnar epithelium was damaged, similarly to the upper respiratory tract (Figure S4).

**Figure 6:**
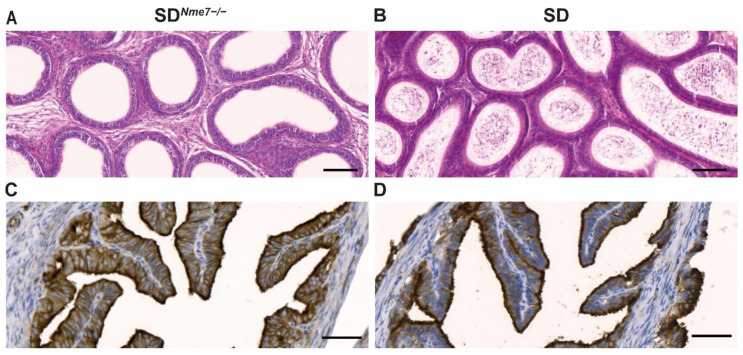
Epididymis (hematoxylin-eosin-stained) of SD*^Nme7−/−^* male rats showing an empty epididymal tubule lumen documenting aspermia (**A**) compared to the normal appearance of sperm in the epididymal tubule lumen in SD control male rats (**B**). Acetylated-α-tubulin staining of a transversally sectioned oviduct in SD*^Nme7−/−^* female rats (**C**) and SD (**D**) female rats. The bars indicate 100 µm for (**A**,**B**), 50 µm for (**C**,**D**).

No sperm was present in the epididymis of SD*^Nme7−/−^* male rats (Figure 6A,B). We observed segments in the oviduct of SD*^Nme7−/−^* female rats showing a damaged simple ciliated columnar epithelium, the predilected site of *Nme7* expression in wild-type rats (Figure S2). In SD rats, acetylated-α-tubulin showed strong positivity in cilia and apical parts of the pseudostratified ciliated epithelium, whereas in SD*^Nme7^*^−/−^ rats, acetylated-α-tubulin staining was evident on the whole surface of ciliated epithelial cells (Figure 6C,D).

### 2.4. Nme7 Expression and Transcriptomic Profile

We corroborated the highest expression of the *Nme7* gene in the testes of wild-type SD rats, followed by lungs, brain, and other tissues, as reported in the data from F344 rats [20] and human expression profiles [21]. The expression of *Nme7* mRNA was over 10 times lower in SD*^Nme7−/−^* testes compared to wild-type SD testes (−10.9 fold; *p* = 1.2 × 10^−10^) and more than three times lower in SD*^Nme7−/−^* lungs compared to wild-type SD lungs (−3.1-fold, *p* = 3.1 × 10^−3^). We performed a western blot to verify the absence of Nme7 protein in the testes. Indeed, we did not detect Nme7 protein in SD*^Nme7−/−^* rats (Figure 1C).

We compared the transcriptomic profiles of lungs (tissue with the highest unisex expression of Nme7 in SD rat) and liver between SD*^Nme7−/−^* and SD rats. The selection of hepatic transcriptome profiling was driven by previous accounts on the involvement of Nme7 in processes related to carbohydrate and lipid metabolism [12,16]. After correcting for multiple comparisons by false discovery rate (FDR < 0.05) and filtering out any transcripts with a smaller than 1.2-fold difference in expression between the respective genotypes, we identified the following numbers of significantly differentially expressed transcripts: 100 in lungs (77 down- and 23 upregulated in SD*^Nme7−/−^* vs. SD rats) and 1552 in the liver (931 down- and 621 upregulated in SD*^Nme7−/−^* vs. SD rats). Only the *Nme7* gene was present in both sets. The complete lists of differentially expressed transcripts are provided in Table S1 (liver) and Table S2 (lungs).

**Figure 7:**
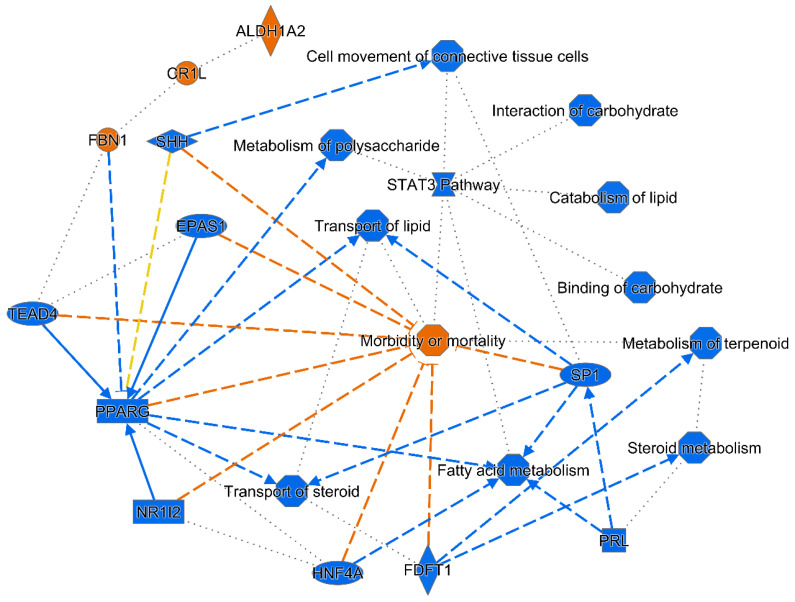
Graphical summary of hepatic transcriptome changes in SD*^Nme7−/−^* rats. The connected subset of the most significant entities within the analysis (upstream regulators, diseases, and biological functions) performed using the machine-learning-based algorithm implemented in the Ingenuity Pathway Analysis is shown. Full and dashed lines correspond to direct and indirect interactions between two entities retrieved from the literature, the inferred edges are shown with dotted lines. The blue color indicates predicted inhibition/downregulation, the orange color indicates predicted activation/upregulation, the yellow lines indicate relationships inconsistent with literature findings for the state of interest.

In the canonical pathway enrichment analysis, we did not observe any statistically significant enrichments in canonical pathways after Benjamini–Hochberg correction in any of the tissues. In the liver, several upstream regulators were identified based on the differentially expressed transcripts, including transforming growth factor beta 1 (Tgfb1; activation z-score—4.00, *p* = 1.5 × 10^−5^), hepatocyte nuclear factor 4 alpha (Hnf4a; activation z-score—3.34, *p* = 9.3 × 10^−5^), and four other upstream regulators were predicted to be inhibited (Table S3A). On the other hand, aldehyde dehydrogenase 1 family member A2 (Aldh1a2) showed the highest activation z-score (2.80, *p* = 4.5 × 10^−4^). A graphical summary presenting a connected subset of the most significant entities within the analysis (upstream regulators, diseases, and biological functions) is shown in Figure 7. The most apparent pattern corresponds to a sharp reduction in lipid and carbohydrate metabolism and transport, combined with decreased cell movement and viability (cell viability not shown in the figure, activation z-score—5.19, *p* = 2.1 × 10^−3^).

In the lungs, detailed analysis showed two enriched disease classes corroborating the histological findings—injury of lung tissue (*p* = 1.59 × 10^−5^) and bleeding of the lung (5.20 × 10^−5^). While no upstream regulators were predicted to be significantly inhibited or activated, the Causal network analysis revealed a potential master regulator protein kinase cAMP-activated catalytic subunit beta (Prkacb), affecting 25 transcripts differentially expressed in SD*^Nme7−/−^* rats compared to wild-type animals (Figure S5). We successfully validated the expression of the subset of the differentially expressed transcripts by qPCR (Figure S6).

## 3. Discussion

The CRISPR/Cas-9-mediated knock-out of the *Nme7* gene in SD rats led to severe hydrocephalus, situs inversus, sinopulmonary disease, infertility of both sexes, and a range of morphological and metabolic dysregulations. Interestingly, disruption of *Nme7* in mice led to situs inversus, only mild hydrocephalus, and no impairment of fertility [18,19], and situs inversus was reported in a family with a homozygous deletion of exon 10 of *Nme7* [17]. These striking features of primary ciliary dyskinesia were present despite the lack of evidence of significant defects in ciliary structure in mice, humans, as well as in our rat model. Hydrocephalus was the most prominent and detrimental feature of SD*^Nme7^*^−/−^ rats, and the rapidity of brain destruction was related to overall survival. Hydrocephalus is defined as an accumulation of cerebrospinal fluid (CSF) within the ventricles of the brain and can be a consequence of different factors. The choroid plexi produce the majority of CSF. Its flow is largely dependent on cilia motility of the ependymal cell lining of the ventricles. In SD*^Nme7^*^−/−^ rats, we detected choroid plexus damage as well as loss of ependymal cell layer postnatally. The first signs of thinning of the neopallial cortex with reduced cellularity in the ventricular zone were observed already at E13.5 stage. The ventricular zone contains radial glia − neural stem cells, that are a mandatory prerequisite for cortical development and the formation of ciliated ependymal cells lining ventricles responsible for CSF movement. Even though the specification of apical radial glial cells in the cortex as ependymal cells starts during embryonal development, their differentiation is not finished until the second week of post-natal life [22]. This corresponds to the timing of external signs of hydrocephalus in the SD*^Nme7^*^−/−^ rats. Such temporal pattern was previously reported as a result of mutation in other cilium-related genes, including *Snx27* and *Hy3* [23]. A decreased number of radial glial cells may also lead to the reduced representation of their downstream lineages of neural and glial cells. CSF was in excessive volumes, pressurized under the skull, and the cortex was the most affected structure of the brain. In rat, CSF is primarily absorbed via the spinal and olfactory routes in both young and old SHRs [24]. The onset and progression of the observed hydrocephalus points to ependymal cell dysfunction, and consequently affected CSF flow, as well as to a subsequent failure of cerebrospinal fluid reabsorption.

The impact of *Nme7* deficiency on the liver was reflected by a robust shift in transcriptome in SD*^Nme7^*^−/−^ rats, with several converging pathways related to pathological processes. Based on the complete dataset, a major predicted upstream node affecting this change were inhibited peroxisome proliferator-activated receptor gamma (*Pparg*) and inferred signal transducer and activator of transcription 3 (*Stat3*) pathways. By far the most affected single transcripts in the liver in SD*^Nme7^*^−/−^ rats were suppressor of cytokine signaling 2 (*Socs2*) and the neuronal regeneration-related protein (*Nrep*), both downregulated more than 15-fold. The latter was recently shown to be a novel molecular mediator of hepatic lipid accumulation and fibrosis development in experimental models and humans by several lines of evidence. Apart from a significant negative correlation of NREP expression with steatosis grade, its lower plasma levels in patients with simple steatosis and nonalcoholic steatohepatitis, NREP is epigenetically modified by parental metabolic syndrome and controls hepatic lipid content [25]. This corresponds with the observed effect on lipid and carbohydrate metabolism pathways in the liver and suppression of Socs2, a critical balancing agent of liver regeneration, proliferation [26], and protection from oxidative stress damage [27]. The fact that we did not observe hepatic steatosis in SD*^Nme7^*^−/−^ rats (Figure S7) could be explained by opposing actions of decreased Nrep favoring lipid deposition and downregulation of *Socs2* and *Pparg*, both of which were shown to prevent hepatic steatosis in spite of negatively affecting insulin resistance [28,29]. The upper and lower respiratory tract pathologies are consistent with the clinical presentation of motile ciliopathies, including rhinitis, sinusitis, and alveolar affection [30,31]. Nme7 is localized both in the cytoplasm and in the nucleus, where it can act as a transcription factor affecting a number of genes in a tissue-specific manner, ultimately contributing to the overall phenotypic landscape beyond its recognized role as a member of the ciliome. There are very little data available so far connecting Nme7 deficiency or variation to overt pulmonary affection. A carrier of the mutant *Nme7* gene showed sinopulmonary symptoms [17], and a suggestive association was found between a loss of CNV involving *Nme7* and equine recurrent airway obstruction [32]. Utilizing Ingenuity Knowledge Base and literature search, we ascertained a set of 197 entities (protein-coding genes, microRNAs) shown to interact with *Nme7* on a molecular level. While our transcriptome data did not directly reveal a mechanistic link between Nme7 loss and the resulting complex pathologies, the identified pathways and nodes may serve as primary candidates for future, more targeted studies. In the lungs, the most downregulated gene *Cpa3* codes for carboxypeptidase 3. It is one of mast cell proteases, recently connected to asthma [33], obesity, and type 2 diabetes [34]. Enhanced expression of adrenomedullin, found in SD*^Nme7^*^−/−^ lungs, was previously reported in patients with asthma and chronic obstructive pulmonary disease (COPD) [35]. Correspondingly, one of the major downregulated nodes was the beta-2-adrenergic receptor, whose agonists are the most widely used agents in treating asthma. Beta-2-adrenergic receptors are expressed throughout the pulmonary system, and their expression positively correlates with lung fluid removal via several distinct mechanisms [36]. Male infertility has long been connected to cilia/flagella-associated genes that are mainly involved in axonemal structure formation [37]. However, a complete lack of sperm in the epididymis, as we observed in SD*^Nme7^*^−/−^ male rats, was reported in studies focusing on genes involved in the development of multiciliated cells such as *Gemc1*, *Mcidas*, and *Ccno* [38]. Mice lacking the aforementioned genes also show female infertility, possibly due to defects of ciliated cells in the oviduct similar to the ones observed in SD*^Nme7^*^−/−^ female rats [39,40,41]. Further studies need to be conducted to elucidate the exact role of Nme7 in male and female infertility.

We acknowledge several limitations of our study. First, given the semi-lethal nature of the induced *Nme7* deficiency, the number of surviving SD*^Nme7^*^−/−^ rats in the post-natal stage available for analysis was limited. On the other hand, the principal reported findings were systematically present in all analyzed animals. A conditional, or tissue-specific knock-out of *Nme7* would be necessary to establish its detailed role in individual organ systems. As in all experimental models, the relevance of our findings to human condition needs to be validated.

In summary, the CRISPR/Cas-9-mediated knock-out of the rat *Nme7* gene resulted in a range of conditions consistent with the presentation of primary ciliary dyskinesia [42], supporting the previously implicated role of the centrosomally located *Nme7* in ciliogenesis and the control of ciliary transport.

## 4. Materials and Methods

### 4.1. Animals

All animal studies were ethically reviewed and performed in accordance with European directive 2010/63/EU and were approved by the Czech Central Commission for Animal Welfare (protocol number 79/2018, approved on 11 September 2018). The Sprague Dawley rats were acquired in-house from a rodent colony kept at the Czech Centre for Phenogenomics, Prague. Rats were housed and handled according to the institutional committee guidelines with free access to food and water. The CRISPR/Cas9 nuclease system was used to generate Sprague Dawley (SD) rats with *Nme7* gene knock-out, as described in detail in the Section 2.1 above.

### 4.2. Genotyping

In the process of derivation of the SD*^Nme7^* line, we established a genotyping protocol, subjecting the isolated genomic DNA to PCR using the primers Nme7R: ‘5′-CCACAGTTAGATGAGGACTAGG-3’ and Nme7F: ‘5′-TGTGTGTCACCACACCCAGC-3’. The PCR product was subsequently cut using TaqI restriction enzyme (Thermo Fisher Scientific, Waltham, MA, USA). Due to the introduced 5-nt (TCGAA) deletion, one TaqI cleavage site is lost; therefore, only 2 fragments are detected in DNA carrying the 5-nucleotide deletion instead of 3 fragments present in SD wild-type rats.

### 4.3. Gene Expression

To assess *Nme7* expression in different organs and tissues of wild-type SD and SD*^Nme7^*^−/−^ rats and to validate the results of transcriptomics, we performed quantitative real-time PCR (RT-qPCR) using used TaqMan probes (Applied Biosystems, Carlsbad, CA, USA) targeting exon boundaries upstream from the deletion in exon 4, between exons 2 and 3 (Rn00593500_m1), and downstream from the deletion in exon 4, between exons 12 and 13 (Rn00684617_m1). Total RNA (1 µg) was reverse-transcribed with oligo-dT primers using the SuperScript IV (Invitrogen, Carlsbad, CA, USA). For validation of the transcriptomic analyses (Supplementary Materials), we used Rn00590969_m1 for caveolin 2 (*Cav2*), Rn00589521_m1 for suppressor of cytokine signaling 2 (*Socs2*), Rn00577779_m1 for hydroxysteroid 17-beta dehydrogenase 2 (*Hsd17b2*), Rn00710306_m1 for insulin-like growth factor 1 (*Igf1*), Rn02132937_s1 for insulin-like growth factor binding protein acid labile subunit (*Igfals*), Rn01462208_m1 for carboxypeptidase A3 (*Cpa3*), Rn01507680_g1 for adrenomedullin (*Adm*). Real-time PCR was performed in triplicate with TaqMan^®^ Gene Expression Master Mix (Applied Biosystems) according to the manufacturer’s protocol (Invitrogen) using the Applied Biosystems^®^ 7900HT Real-Time PCR System. Results were analyzed using the Livak analysis method [43] 30, with glyceraldehyde 3-phosphate dehydrogenase (*Gapdh*) as a reference gene.

### 4.4. Transcriptome Profiling

Total RNA was isolated from the liver and lung tissues (RNeasy Mini Kit, Qiagen, Hilden, Germany) of SD and SD*^Nme7^*^−/−^ rats. The quality and integrity of the total RNA were evaluated on an Agilent 2100 Bioanalyzer system (Agilent, Palo Alto, CA, USA). Microarray experiments were performed using the Rat Gene 2.1 ST Array Strip in 4 animals per group. The whole hybridization procedure was performed using the Affymetrix GeneAtlas^®^ system according to the manufacturer’s instructions. The quality control of the chips was performed using Affymetrix Expression Console. After applying quality filters and data normalization by the Robust Multichip Average (RMA) algorithm implemented in Affymetrix Expression Console, the set of obtained differentially expressed probesets was filtered by the FDR method implemented in PARTEK Genomics Suite 7 (Partek, St. Louis, MO, USA). Transcriptomic data were then processed by a standardized sequence of analyses (hierarchical clustering and principal component analysis, gene ontology, gene set enrichment, Upstream Regulator Analysis, Mechanistic Networks, Causal Network Analysis, and Downstream Effects Analysis) using Ingenuity Pathway Analysis [44].

### 4.5. Western Blot

We used a commercially available Nme7 antibody (ab220753, Abcam, Cambridge, MA, USA) and proceeded as follows: on a 12% SDS-PAGE gel, we loaded 10 μL of sample solution at a 2500 ng/μL concentration in each well. After electrophoresis, the resolved proteins were transferred onto PVDF membranes. The membranes were incubated with the Nme7 antibody at a dilution of 1:6000 overnight at 4 °C. The membranes were subsequently washed three times for 10 min with TBS buffer, and then, a secondary HRP-conjugated antibody (Amersham Little Chalfont,) was used, and signal was detected using the ECL Prime chemiluminescent detection kit (GE Healthcare Bio-Sciences, Little Chalfont, UK) and Hyperfilm ECL. The developed hyperfilms were scanned, and densitometry performed in ImageJ [45]. The theoretical molecular weight of unmodified Nme7 is 44.5 kDa. The observed signal was consistent with the expected value.

### 4.6. Necropsy and Histology

We performed a necropsy and histological assessment of both SD*^Nme7^*^−/−^ male and female rats (*n* = 17; 7 pre-natal, 10 post-natal). At the age of 6 weeks, the following organs were sampled: adrenal gland, heart, mammary gland (in female rats only (F)), skin, thymus, brain, kidney, ovary (F), small bowel, thyroid, epididymis (in male rats only (M)), large bowel, pancreas, spinal cord, trachea, esophagus, liver, prostate (M), spleen, urinary bladder, eye, lungs, seminal vesicles (M), stomach, uterus (F), lymph node, skeletal muscles, adipose tissue, and testis (M). Sampled organs were fixed for 24 h in a 10% formalin solution and processed by standard histological methods using an automated tissue processor (Leica ASP6025, Leica Microsystems, Wetzlar, Germany) and then embedded in paraffin blocks using a Leica EG 1150H paraffin embedding station (Leica Microsystems, Wetzlar, Germany). Three to five μm-thick slices were cut from each sample using a microtome (Leica RM2255, Leica Microsystems, Germany) and mounted on standard glass slides (Bamed, Litvínovice, Czech Republic). The first slices were stained with hematoxylin–eosin (DiaPath, Martinengo, Italy) using Ventana Symphony H&E Slide Stainer (Ventana Medical Systems, Inc., Tucson, AZ, USA). The prepared samples were evaluated as light microscopic images obtained using a Carl Zeiss Axio Scope A1 (Zeiss, Oberkochen, Germany) and the Axio Scan.Z1 slide scanner (Zeiss, Germany). Additional histological analyses were performed on rat embryos obtained from uteri of pregnant female rats of all three genotypes at E13.5, 14.5, and 15.5. Isolated embryos were also imaged using a Zeiss AxioZoom (Carl Zeiss Microscopy GmbH, Oberkochen, Germany) macroscope. Immunohistochemistry was performed in tissue samples that showed signs of pathology in SD*^Nme7^*^−/−^ rats utilizing hematoxylin–eosin staining. We used commercially available antibodies (Abcam, Cambridge, MA, USA) against Nme7 (ab220753), acetylated α-tubulin (ab24610), γ-tubulin (for Western blot, ab11321), and secondary antibodies HRP-conjugated goat-anti-rabbit (ab205718), according to manufacturer’s instructions.

### 4.7. Micro-CT

Whole-mount postnatal rats were euthanized and fixed for 7 days in 4% PFA. Samples were then processed for micro-CT scanning including incubation in contrast agent. Samples were stained for at least 14 days with Lugol’s Iodine solution. The stock solution (10 g KI and 5 g I_2_ in 100 mL H_2_O) was diluted to a 25% working solution in H2O to achieve neutral osmotic pressure and avoid tissue distortion. A SkyScan 1176 microCT (Bruker, Kontich, Belgium) was set up for voxel size 9 µm and 1 mm Al filter. A 360° scan with 0.200° rotation step and 3 frames averaging setup was used for scanning.

### 4.8. Transmission Electron Microscopy (TEM)

For TEM imaging, tissues were fixed in 2% glutaraldehyde and 2% paraformaldehyde at room temperature for 2 h, rinsed in 1× PBS, post-fixed with 1% OsO_4_ at room temperature for 2 h, and washed in 1× PBS overnight. Next, tissues were dehydrated in an alcohol, substituted with propylene oxide, and embedded in the Epon mixture. Sections were collected on formvar/carbon-coated nickel grids and visualized by TEM (FEI Morgagni).

### 4.9. Statistical Analysis

Statistical analyses were performed using STATISTICA 13.5 (TIBCO, Germany) for comparison of morphometric and metabolic variables between SD*^Nme7^*^−/−^ and SD rats (Student’s *t*-test). The null hypothesis was rejected whenever *p* > 0.05. In transcriptome assessment, the correction for multiple comparisons was performed by applying FDR < 0.05 in PARTEK Genomics Suite, followed by filtering out transcripts with expression difference less or equal to 1.2-fold in the particular tissue between SD*^Nme7^*^−/−^ and SD rats. Only transcripts passing these criteria were then subjected to analyses in Ingenuity Pathways Analysis described above, where Benjamini–Hochberg multiple testing correction was applied for Upstream Regulator, Causal Network, Canonical Pathway, Disease or Function analyses [44].

## Data Availability

The datasets generated during and/or analyzed during the current study are available from the corresponding author on reasonable request. The microarray data generated during and/or analyzed during the current study are available in the ArrayExpress repository (http://www.ebi.ac.uk/arrayexpress/experiments/E-MTAB-10000, accessed on 6 April 2021).

## Supplementary Materials

The following are available online at https://www.mdpi.com/article/10.3390/ijms22083810/s1, Figure S1: Gross morphology of SD*^Nme7^*^−/−^ and SD embryos, Figure S2: Immunohistochemical detection of Nme7 in trachea and oviduct of SD*^Nme7^*^−/−^ and SD rats, Figure S3: Paranasal sinuses of SD*^Nme7^*^−/−^ rat with mucinous deposits, Figure S4: Hematoxylin–eosin- and acetylated-α-tubulin-stained lungs of SD*^Nme7^*^−/−^ and SD rats, Figure S5: Top resulting network from the Causal Network Analysis algorithm based on significantly differentially expressed transcripts in the lungs of SD*^Nme7^*^−/−^ vs. SD rats, Figure S6: Validation of the transcriptomic results by qPCR, Figure S7: Hematoxylin–eosin-stained liver of SD*^Nme7^*^−/−^ and SD rats, Table S1: List of genes significantly differentially expressed in the liver of SD*^Nme7^*^−/−^ vs. SD rats, Table S2: List of genes significantly differentially expressed in the lungs of SD*^Nme7^*^−/−^ vs. SD rats, Table S3: List of upstream regulators predicted to be activated or inhibited based on the transcriptome changes in the liver, Video S1: Video showing morphological and behavioral differences between SD*^Nme7^*^−/−^ and SD two-week-old rat littermates.
