# Supplementary material for: Semi-Lethal Primary Ciliary Dyskinesia in Rats Lacking the Nme7 Gene
Source: Int J Mol Sci. 2021 Apr 7;22(8):3810. doi: 10.3390/ijms22083810 (PMC8067621; doi:10.3390/ijms22083810)
Supplement: Supplementary file 1 [file ijms-22-03810-s001.zip › SupplementaryMaterial_Figures.pdf]

Supplementary Material - Figures

Semi-lethal primary ciliary dyskinesia in rats lacking *Nme7* gene

Lucie Šedová [1,2,*], Ivana Buková [5], Pavla Bažantová [2], Silvia Petrezsélyová [1,5], Jan Prochazka [5], Elena Školníková [1,2], Dagmar Zudová [5], Josef Včelák [3], Pavol Makovický [4], Běla Bendlová [3], Ondřej Šeda [2] and Radislav Sedlacek [1,5]

[1] Laboratory of Transgenic Models of Diseases, Institute of Molecular Genetics of the Czech Academy of Sciences, v.v.i., Vestec, Czech Republic;
[2] Institute of Biology and Medical Genetics, the First Faculty of Medicine, Charles University and the General University Hospital, Prague, Czech Republic;
[3] Department of Molecular Endocrinology, Institute of Endocrinology, Prague, Czech Republic;
[4] Department of Biology, Faculty of Education, J. Selye University, Komarno, Slovak Republic;
[5] Czech Centre for Phenogenomics, Institute of Molecular Genetics of the Czech Academy of Sciences, v.v.i., Vestec, Czech Republic;
* Correspondence: lucie.sedova@lf1.cuni.cz

Supplementary Figure S1

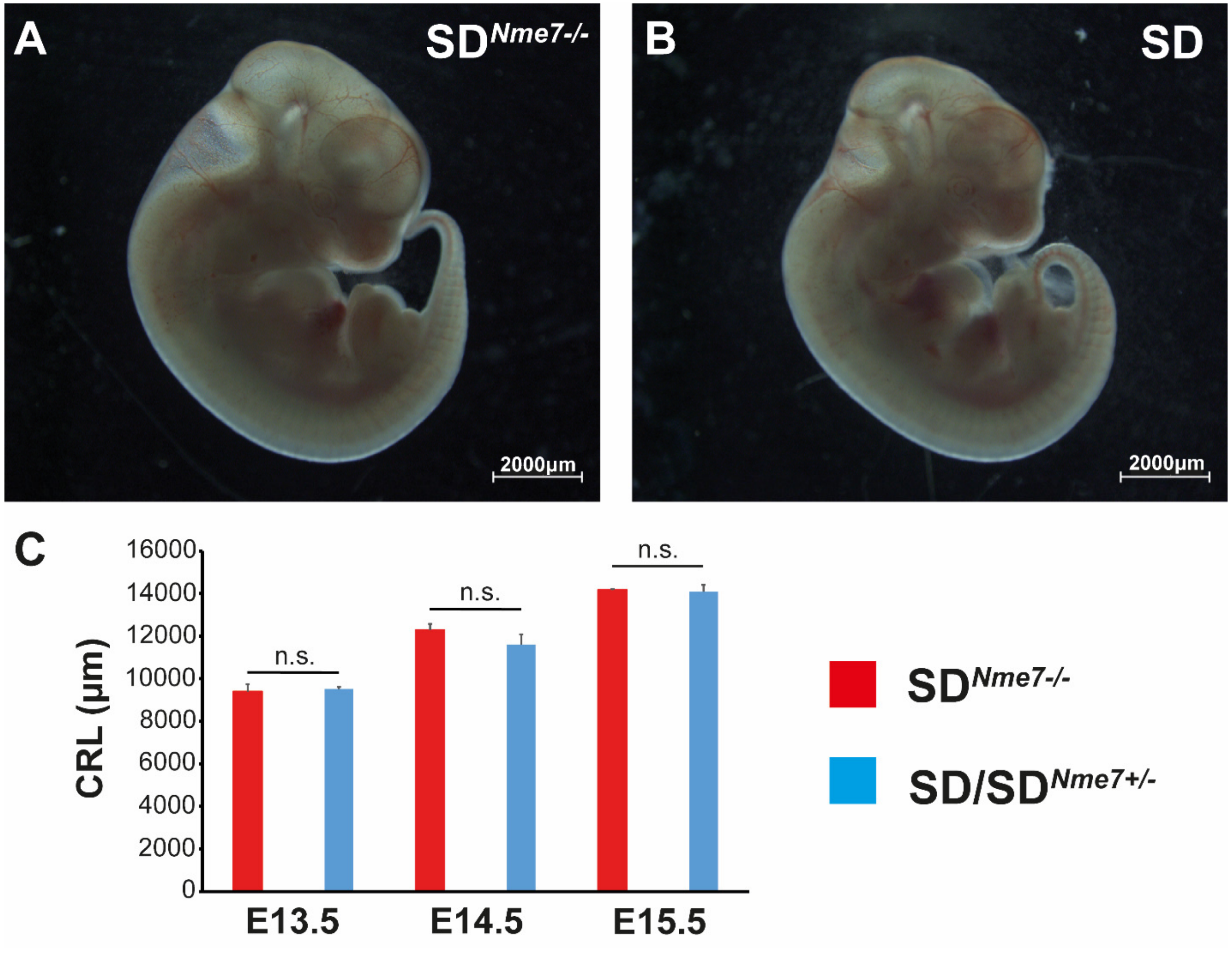

Figure S1. Gross morphology of SD$^{Nme7-/-}$ (**A**) and SD embryos (**B**) at E13.5. **C**. The crown-to-rump length (CRL) measurement did not show any differences between SD$^{Nme7-/-}$ and SD / SD$^{Nme7+/-}$ embryos at E13.5, E14.5, and E15.5.

Supplementary Figure S2

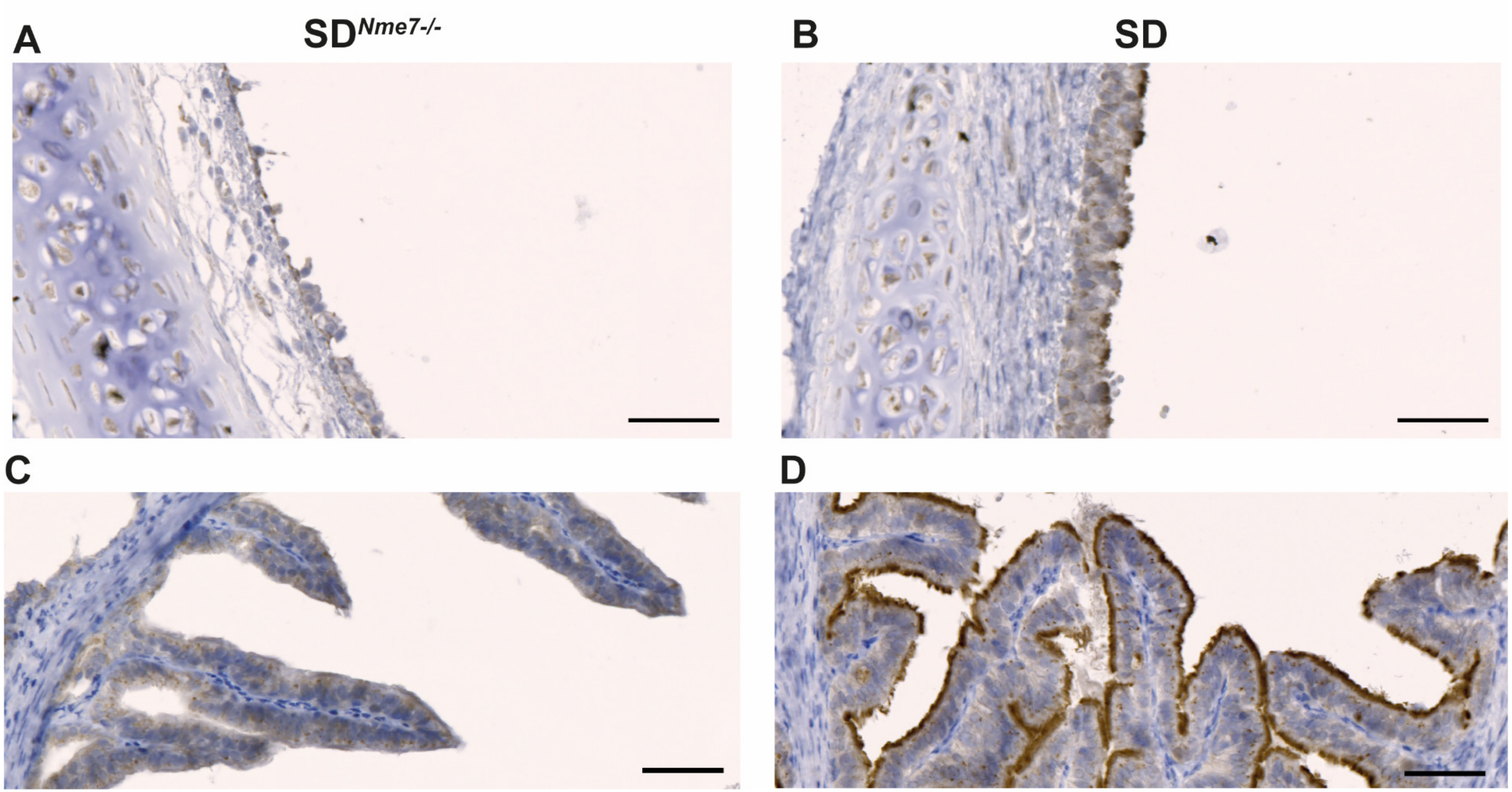

Figure S2. Expression of Nme7 was detected using immunohistochemistry in transversally sectioned trachea (**A, B**) and oviduct (**C, D**). In contrast to damaged, almost missing pseudostratified ciliated columnar epithelium in SD$^{Nme7-/-}$ (**A**), the Nme7 showed positivity in pseudostratified ciliated columnar epithelium in SD rat (**B**). Transversally sectioned oviduct with folded pseudostratified ciliated epithelium lining showing strong positivity for Nme7 in SD control female rat (**D**), contrasting with damaged pseudostratified ciliated epithelium in SD$^{Nme7-/-}$ female rat (**C**). The bars indicate 50 μm.

Supplementary Figure S3

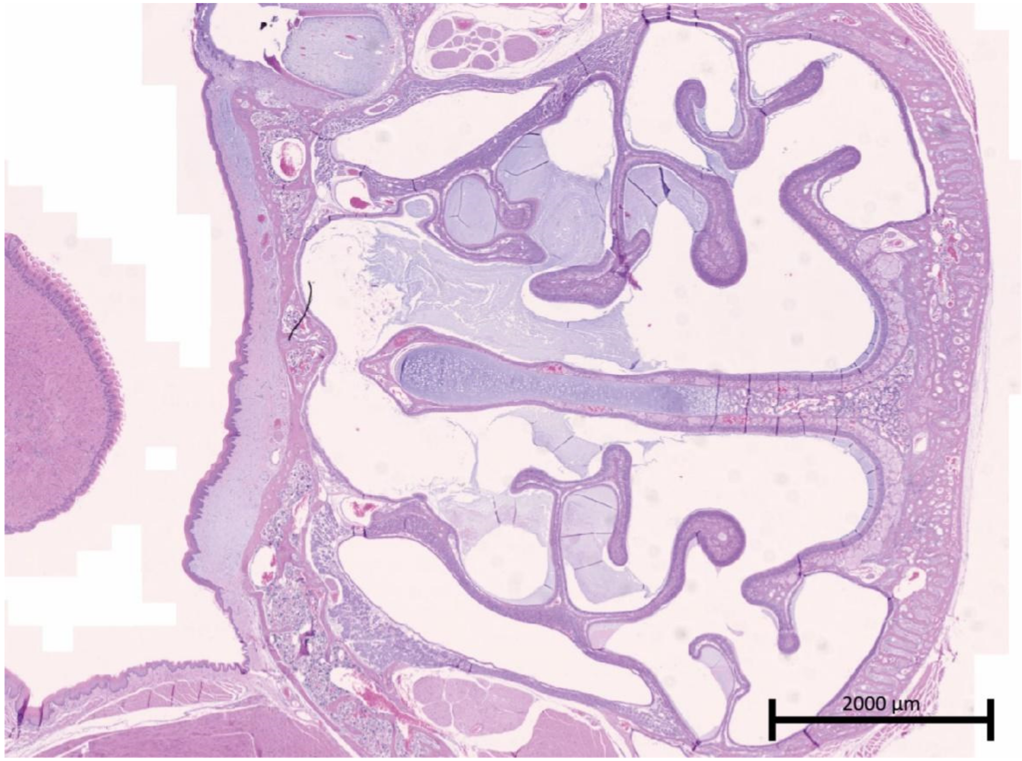

Figure S3. Paranasal sinuses (haematoxylin-eosin stained) of SD$^{Nme7-/-}$ rat with mucinous deposits. The bar indicates 2000 µm.

Supplementary Figure S4

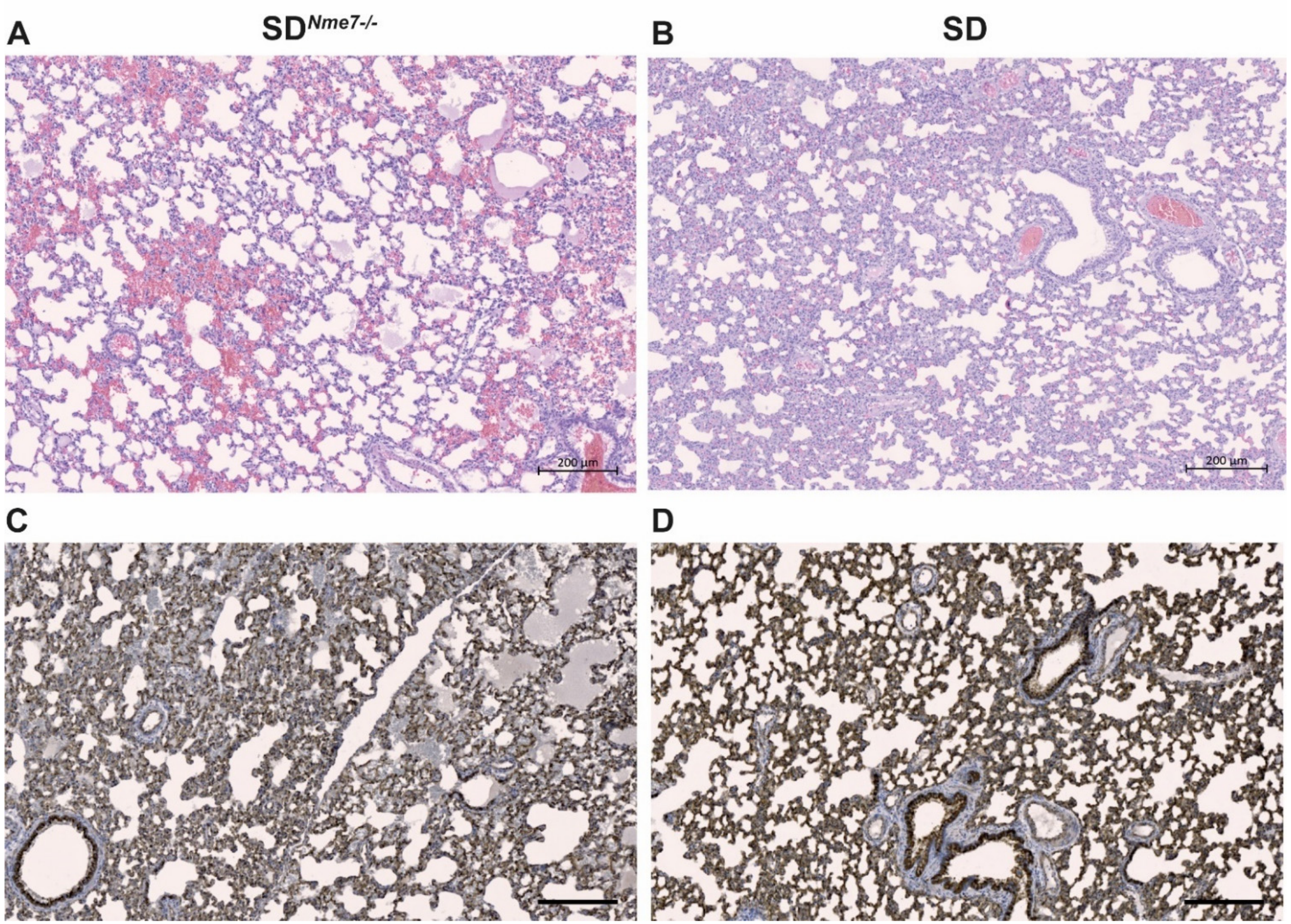

Figure S4. Haematoxylin-eosin stained lungs in $SD^{Nme7-/-}$ (**A**) and SD (**B**) rats. Acetylated-α-tubulin staining of lungs in $SD^{Nme7-/-}$ (**C**) and SD (**D**) rats. The bars indicate 200 µm.

Supplementary Figure S5

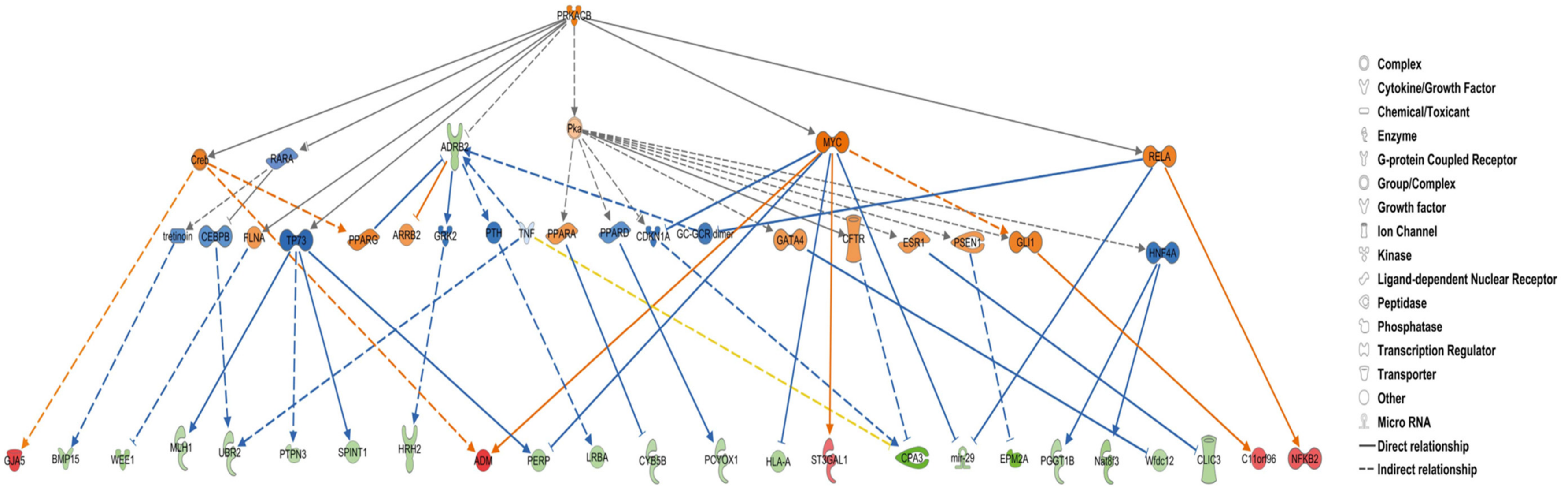

Figure S5. Top resulting network from Causal Network Analysis algorithm based on significantly differentially expressed transcripts in the lungs of SD$^{Nme7-/-}$ vs. SD rats. Causal network combining several layers of upstream regulators indicating their predicted activation (shades of orange) or inhibition (shades of blue) in the lungs of SD$^{Nme7-/-}$ rats compared to their SD littermates. The transcripts significantly differently expressed between SD$^{Nme7-/-}$and SD are shown in shades of green (downregulation in SD$^{Nme7-/-}$) and shades of red (upregulation in SD$^{Nme7-/-}$). Network was generated using Causal networks Analysis within Ingenuity Pathway Analysis (Qiagen) Advanced Analytics module.

Supplementary Figure S6

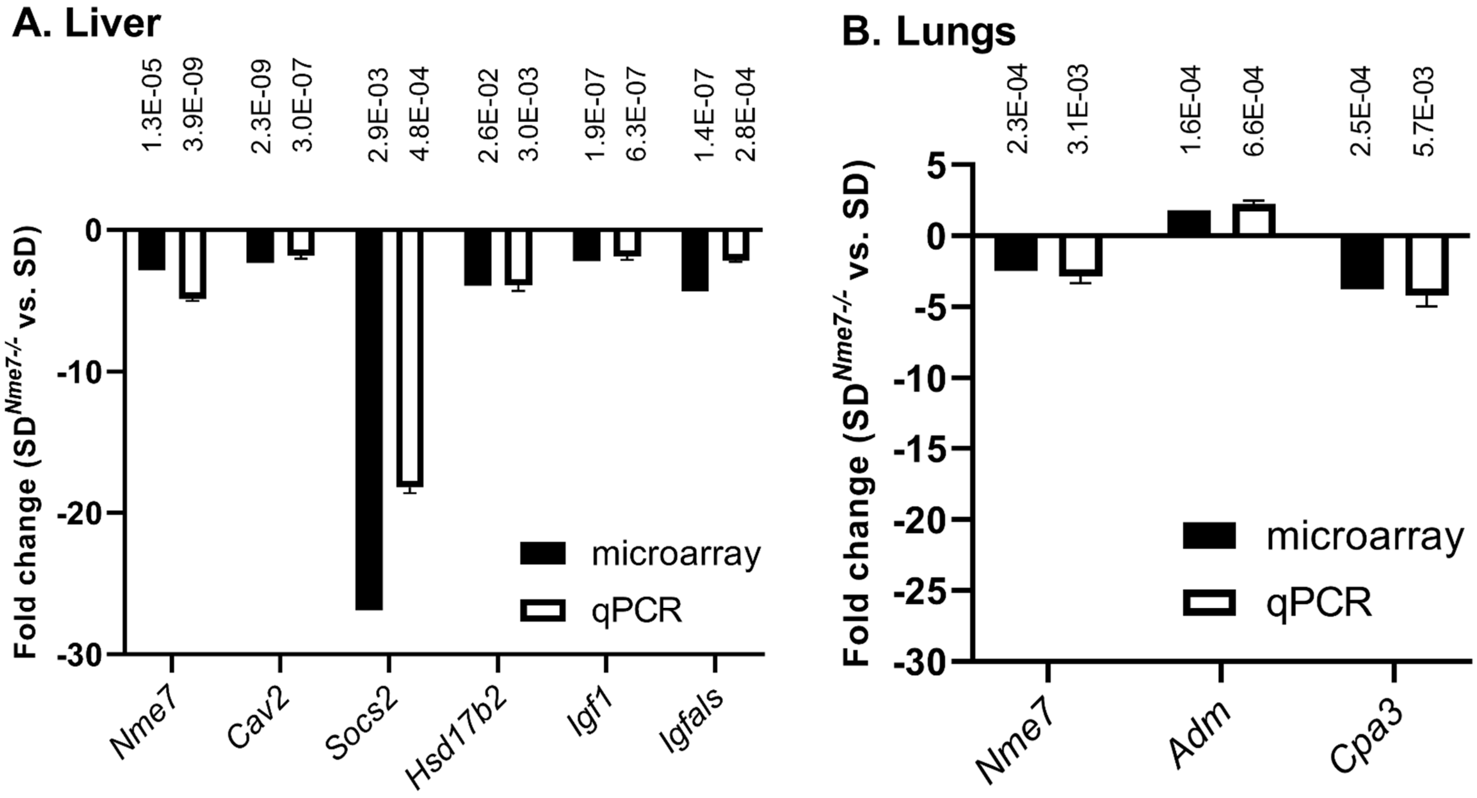

Figure S6. Validation of transcriptomic results by qPCR. Fold changes are indicated for comparison of expression between SD$^{Nme7-/-}$ vs. SD rats (qPCR: blue bars; microarray: red bars) in **A**. liver; **B**. lungs. The microarray fold-change values are shown based on Partek Genomics Suite analysis (see Methods); the qPCR results are expressed as the mean fold-change + standard error of the mean for each transcript. The indicated significance levels correspond to FDR-corrected p-value for microarray (Partek) and the two-sample t-test for qPCR. The gene symbols are used in accordance with the names approved by the HUGO Gene Nomenclature Committee: caveolin 2 (*Cav2*), suppressor of cytokine signaling 2 (*Socs2*), hydroxysteroid 17-beta dehydrogenase 2 (*Hsd17b2*), insulin-like growth factor 1 (*Igf1*), and insulin-like growth factor binding protein acid labile subunit (*Igfals*), adrenomedullin (*Adm*), carboxypeptidase A3 (*Cpa3*).

Supplementary Figure S7

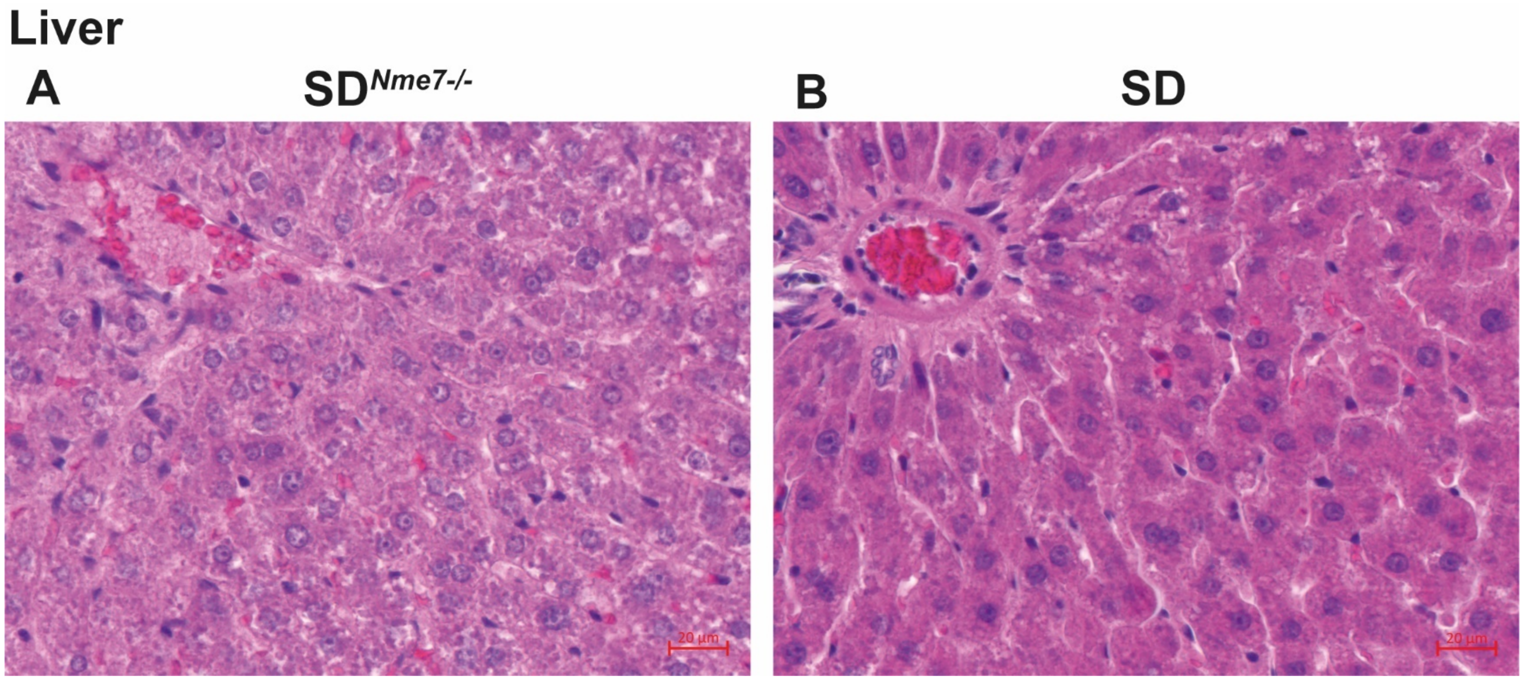

Figure S7. Haematoxylin-eosin stained liver in SD$^{Nme7-/-}$ (**A**) and SD (**B**) male rats.
